# Alcohol-and-HIV-Induced Lysosomal Dysfunction Regulates Extracellular Vesicles Secretion *in Vitro* and in Liver-Humanized Mice

**DOI:** 10.3390/biology10010029

**Published:** 2021-01-05

**Authors:** Raghubendra Singh Dagur, Moses New-Aaron, Murali Ganesan, Weimin Wang, Svetlana Romanova, Srivatsan Kidambi, Kusum K. Kharbanda, Larisa Y. Poluektova, Natalia A. Osna

**Affiliations:** 1 Research Service, Veterans Affairs Nebraska-Western Iowa Health Care System, Omaha, NE 68105, USA; moses.newaaron@unmc.edu (M.N.-A.); murali.ganesan@unmc.edu (M.G.); kkharbanda@unmc.edu (K.K.K.); 2 Department of Internal Medicine, University of Nebraska Medical Center, Omaha, NE 68198, USA; 3 Department of Environmental, Agriculture and Occupational Health, College of Public Health, University of Nebraska Medical Center, Omaha, NE 68102, USA; 4 Department of Pharmacology and Experimental Neuroscience, University of Nebraska Medical Center, Omaha, NE 68198, USA; Weimin.Wang@unmc.edu (W.W.); lpoluekt@unmc.edu (L.Y.P.); 5 Department of Pharmaceutical Sciences, University of Nebraska Medical Center, Omaha, NE 68198, USA; sromanova@unmc.edu; 6 Department of Chemical and Biomolecular Engineering, University of Nebraska, Lincoln, NE 68588, USA; skidambi2@unl.edu

**Keywords:** small EVs, humanized mice, ethanol metabolism, liver disease, next-generation RNA sequencing

## Abstract

**Simple Summary:**

Consumption of alcohol increases liver damage in HIV people; however, the mechanisms remain elusive. By utilizing alcohol (ethanol) exposed HIV-infected human hepatocytes liver cells and ethanol-fed liver humanized mouse model (immunodeficient mice livers harbor human hepatocytes), we show that the combination of alcohol and HIV enhances oxidative stress and impairs lysosome activity, which in turn stimulates pathogenic nanosized extracellular vesicles. These vesicles are of great importance, as manipulating their numbers and contents will eliminate the spread of infection in other cell types.

**Abstract:**

Background: Alcohol abuse is common in people living with HIV-1 and dramatically enhances the severity of HIV-induced liver damage by inducing oxidative stress and lysosomal dysfunction in the liver cells. We hypothesize that the increased release of extracellular vesicles (EVs) in hepatocytes and liver humanized mouse model is linked to lysosome dysfunction. Methods: The study was performed on primary human hepatocytes and human hepatoma RLW_XP-GFP_ (Huh 7.5 cells stably transfected with CYP2E1 and XPack-GFP) cells and validated on ethanol-fed liver-humanized fumarylacetoacetate hydrolase (Fah)^-/-^, Rag2^-/-^, common cytokine receptor gamma chain knockout (FRG-KO) mice. Cells and mice were infected with HIV-1_ADA_ virus. Results: We observed an increase in the secretion of EVs associated with a decrease in lysosomal activity and expression of lysosomal-associated membrane protein 1. Next-generation RNA sequencing of primary human hepatocytes revealed 63 differentially expressed genes, with 13 downregulated and 50 upregulated genes in the alcohol–HIV-treated group. Upstream regulator analysis of differentially expressed genes through Ingenuity Pathway Analysis identified transcriptional regulators affecting downstream genes associated with increased oxidative stress, lysosomal associated disease, and function and EVs biogenesis. Our in vitro findings were corroborated by in vivo studies on human hepatocyte-transplanted humanized mice, indicating that intensive EVs’ generation by human hepatocytes and their secretion to serum was associated with increased oxidative stress and reduction in lysosomal activities triggered by HIV infection and ethanol diet. Conclusion: HIV-and-ethanol-metabolism-induced EVs release is tightly controlled by lysosome status in hepatocytes and participates in the development of double-insult-induced liver injury.

## 1. Introduction

Despite significant improvement in the survival of people living with HIV-1 (PLWH) in the era of highly efficient antiretroviral therapy, liver disease is emerging as a major cause of morbidity and mortality [1]. Approximately 48% of PLWH have a history of alcohol abuse [2]. Alcohol potentiates HIV-mediated liver damage, and alcohol consumption in PLWH exacerbates liver injury, leading to hepatic fibrosis or cirrhosis. It also enhances pathological infection features by increasing viremia, suppressing the immune response, and promoting non-adherence to treatment, resulting in poor HIV treatment outcomes [3,4]. Alcohol also interferes with the biogenesis and lysosomal activity and may contribute to accumulation or subjected to degradation of proteins (including HIV proteins) in the liver, which may have harmful hepatotoxic effects [5]. Our group’s previous study demonstrated that ethanol metabolism potentiates the accumulation of HIV proteins/HIV RNA and causes oxidative stress and lysosome impairment in the hepatocytes [5].

As demonstrated before, in alcoholic liver disease, human hepatocytes release significant amounts of extracellular vesicles (EVs), a typical feature of alcoholic hepatitis [6]. HIV interaction with multiple hepatic cell types, such as Kupffer cells (KCs) and hepatic stellate cells (HSCs), has been reported to impair intercellular communication networks for supporting liver homeostasis [7], attributing to an increased EVs release [8]. Endosome-origin exosomes are excreted from the cells when multivesicular bodies (MVBs) of the endosomal system fuse with the plasma membrane. However, the mechanisms to sort MVBs, either to the plasma membrane or the lysosome, are unclear. Furthermore, a checking point between these two fates suggests that one pathway’s inhibition may promote the switch to another pathway [9,10]. It has also been shown that lysosome inhibition with various chemicals, such as bafilomycin A1, increases EV secretion [11]. EVs may contain regulatory molecules, which represent lysosome non-degraded cell products, and this degradation is suppressed by ethanol (EtOH) metabolism in hepatocytes [5]. The role of EVs in the context of ethanol and HIV-1 from cells other than hepatocytes is well reported [12,13]. However, the combined effects of HIV and ethanol metabolism on the EVs release and EVs secretion association with lysosomal activity in hepatocytes have not been examined.

Here, we hypothesize that the combination of HIV infection and ethanol metabolism causes lysosome dysfunction in hepatocytes, thereby potentiating EVs release from these cells. Our findings indeed indicate that, in HIV-infected hepatocytes, ethanol metabolites and, mainly, acetaldehyde (Ach) upregulate the expression of stress-related genes, leading to lysosome dysfunction and a subsequent increase in EVs secretion from hepatocytes.

## 2. Materials and Method

### 2.1. Reagents

Reagents were obtained from the following commercial sources: high-glucose Dulbecco’s Modified Eagle Medium (DMEM), William’s E Medium with plating and maintenance supplements, penicillin–streptomycin, and LysoTracker™ Red DND-99 (Thermofisher, Carlsbad, CA, USA); LysoSensor^TM^ Yellow/Blue DND-160 (PDMPO) (Thermofisher, Carlsbad, CA, USA); fetal bovine serum (FBS) (R&D Systems, Minneapolis, MN, USA); human pooled serum (MP Biomedicals, Irvine, CA, USA); Puromycin (Invivogen, San Diego, CA, USA); ExoQuick-TC exosome precipitation solution (SBI, Palo Alto, CA, USA); primary antibodies- Alix TSG 101, CD9, LAMP1, cathepsin D, β-actin, and calnexin (Santa Cruz, Santa Cruz, CA, USA), HIV p24 (Abcam, Boston, MA, USA); human pan-exosome isolation kit (Miltenyi Biotec Inc, San Francisco, CA, USA); collagenase (Worthington Biochemical Corporation, Lakewood, NJ, USA); and TBARS (Thiobarbituric acid reactive substances) assay kit (Cayman Chemicals, Ann Arbor, MI, USA). Bafilomycin A1, chloroquine, N-Acetyl-L-cysteine (NAC), and all analytical-grade quality reagents were from Sigma (St. Louis, MO, USA).

### 2.2. Cells and Treatments

Primary human hepatocytes (PHH) were obtained from the liver tissue distribution system (Minneapolis, MN; Pittsburgh, PA; NIH Contract #HSN276201200017C) and maintained in serum-free William’s E Medium with maintenance supplement. Hepatocytes were plated on polyelectrolyte multilayer (PEM) film coating on top of the polydimethylsiloxane surface (two-dimensional (2D) culture) to support a long-term cell functionality since cells plated on collagen undergo fast de-differentiation and lose expression of ethanol metabolizing enzymes, cytochrome P4502E1 (CYP2E1), and alcohol dehydrogenase (ADH) in 24 h [14,15]. Thereby, PHH was exposed to 50 mM EtOH treatment for 24 h, followed by HIV infection, as published earlier [5]. Due to uncertainty in human hepatocytes’ availability, we also used human Huh7.5^CYP2E1^ (RLW) cells [5]. RLW cell line metabolizes ethanol by CYP2E1 (catalyzing reactive oxygen species (ROS) formation), but do not express ADH, the primary source of acetaldehyde, Ach. These cells were also transduced with XPack CMV-XP-GFP-EF1α-Puro expression lentivector, following the manufacturer’s instruction (XPAK530PA-1, System Biosciences, Palo Alto, CA, USA), to produce GFP-labeled EVs [16]. Transduced cells were cultured in DMEM medium containing puromycin 1.0 μg/mL for the selection of puromycin-resistance cells, to make a stable cell line and to minimize the GFP artifact. Furthermore, GFP-positive cells (RLW_XP-GFP_) were purified by using fluorescence-activated cell sorting and imaged on fluorescence microscopy (Supplementary Materials Figure S1a). RLW_XP-GFP_ cells expressing GFP were comparable to parent RLW cells in viability and growth kinetics (Supplementary Materials Figure S1b,c). Because these cells do not produce Ach, we exposed them to the acetaldehyde-generating system (AGS). AGS contains yeast ADH as a source of enzyme, nicotinamide adenine dinucleotide as a co-factor, and 50 mM EtOH (substrate for ADH), and it continuously produces physiologically relevant amounts of Ach without toxic effects. AGS treatment in ADH-non-expressing RLW cells recapitulates the effects of ethanol on ethanol-metabolizing hepatocytes. The transduced RLW_XP-GFP_ cells were exposed to AGS, while PHH were exposed to 50 mM EtOH and infected with HIV_ADA_ at the multiplicity of infection (MOI) 0.1 for 24 h [17]. After overnight HIV-infection, cells were washed with phosphate-buffered saline (PBS) four times to remove the free virus and incubated in a serum-free medium for an additional 24 h, and for 72 h to induce death and establish correlation with EVs release. For lysosome inhibition and alkalization, RLW_XP-GFP_ cells were treated with varying concentrations of bafilomycin A1 for four hours and chloroquine for overnight. In some experiments, cells were treated with 5 mM N-acetyl cysteine (NAC) antioxidant (dissolved in ddH2O, pH 7.4) applied 1 h before AGS treatment. Lysosomes were stained by incubating cells with 50 nM LysoTracker red DND-99 followed by mounting of coverslips with ProLong™ Gold Antifade mountant with DAPI. The images were acquired on a Confocal LSM710 microscope and quantified by using Fiji [18]. The pH of acidic organelles, such as lysosome in RLW_XP-GFP_ cells, was assessed by using LysoSensor™ Yellow/Blue DND-160 (Thermofisher, Carlsbad, CA, USA), that produces blue fluorescence in neutral environments and used as per manufacturer’s instructions. Briefly, live cells were incubated with 1 μM LysoSensor Yellow/Blue DND-160 in the pre-warmed medium for 5 min at 37 °C. After washing with a probe-free medium, the samples were viewed with an EVOS fluorescence microscope (Thermofisher, Carlsbad, CA, USA).

### 2.3. Experimental Manipulations on FRG-KO Mice with Liver Humanization

Fumarylacetoacetate hydrolase (Fah)^-/-^, Rag2^-/-^, common cytokine receptor gamma chain knockout (FRG-KO; C57BL/6 strain background) male mice transplanted with human hepatocytes from a single donor (>80% humanization) were purchased from Yecuris Corporation (Tualatin, OR, USA) and housed in the pathogen-free animal facility at the University of Nebraska Medical Center (UNMC). Animal studies were carried out according to the guidelines for the humane care of laboratory animals as approved by the UNMC Animal Care and Use Committee (IACUC 19-034-05FC). Human-specific albumin levels were assessed in the serum of humanized mice with an ELISA kit (Bethyl Laboratories Inc., Montgomery, TX, USA), as described previously [19]. These mice showed average human albumin levels of 7161 ± 276 μg/mL. The mice were randomized into four groups and were pair-fed with isocaloric control or 5% EtOH diets, using the NIH-Gao model [20] combined or not with HIV infection. Each group, namely control, HIV, EtOH, and EtOH–HIV, contained three mice. For intraperitoneal injections, HIV-1_ADA_ was injected at a dose of 10^5^ TCID_50_/mouse every third day (a total of three injections during the feeding period). Control uninfected mice received sham-injection with saline. After two days of the last HIV injection, blood was drawn, and mice were euthanized to harvest livers. Non-humanized mice served as a negative control for liver humanization.

### 2.4. OptiPrep Method for EVs Separation from In Vitro Cultures

All EV isolation and characterization procedures corresponded to minimal information for studies of extracellular vesicles 2018 (MISEV2018) guidelines [21]. A post-treatment serum-free conditioned medium was collected from an equal number of plated cells, centrifuged at low-speed (300× *g*) for 10 min, to remove cells, and then at 2000× *g* for 10 min at 4 °C, to eliminate dead cells and debris. Supernatants were then filtered through 0.22 μm filters and centrifuged at 10,000× *g* for 30 min to exclude large vesicles and further ultra-centrifuged at 100,000× *g* for 70 min at 4 °C, using SW28 Ti (k-factor: 245) or SW40Ti (K factor: 137) rotors (Beckman, Palatine, IL USA) to concentrate small EVs. In some cases, where the supernatant’s volume was less than 7 mL, EVs were isolated by using ExoQuick-TC precipitation solution, per manufacturer instructions. To further separate EVs that contain HIV, EV pellets were further resuspended in 1 mL of 1x PBS, and top-loaded on PBS diluted 6–18% iodixanol (OptiPrep; Cosmo Bio, Carlsbad, CA, USA) with an increment of 1.2% as described previously [22]. Gradient fractions were collected from the top of the gradient in 1 mL increments and transferred to sterile 1.5 mL centrifuge tubes. Gradients were ultracentrifuge at 100,000× *g*, using SW41 Ti rotor for 18 h at 4 °C, and then 12 fractions containing 1 mL were taken from the top of the gradient (named F1–F12), diluted in PBS, and pelleted again for one hour at 100,000× *g* in an SW41 Ti rotor to get rid of iodixanol from the samples. These fractions were characterized to demonstrate EVs’ presence with markers: transmembrane or GPI-anchored proteins CD9; cytosolic proteins recovered in EVs, Alix and TSG101; and small EVs (sEVs; size < 200 nm) negative marker calnexin. Cell lysate from uninfected RLW_XP-GFP_ cells was used as a negative control to viral protein p24. Alternatively, HIV-free OptiPrep fractions F2–F8 were pooled and resuspended in sterile 1 x PBS, followed by ultracentrifugation for 70 min at 100,000× *g*, using SW41 Ti rotor to pellet EVs for further experiments.

### 2.5. Isolation of EVs from Liver

EVs were isolated by using differential centrifugation, ultracentrifugation, precipitation methods and purified as described previously with slight modifications [23]. Harvested livers from each mouse group were weighed, and an equal amount was used for isolation of EVs. Livers were homogenized in 10 mL of activating solution consisting of PBS with 1% collagenase enzyme and incubated at 37 °C for ~30 min on gentleMACS Octo Dissociator with heaters (Miltenyi Biotec, San Francisco, CA, USA), using inbuilt 37C_m_LIDK_1 program. Following homogenization, the suspension was filtered through 40 μm filters, centrifuged at 300× *g* for 10 min, and then at 2000× *g* for 10 min at 4 °C, to eliminate tissue fragments, cells, dead cells, and debris, respectively. Supernatants were then filtered through 1 μm filters and further centrifuged at 10,000× *g* for 30 min, to discard unwanted large vesicles. The supernatant was then filtered through 0.45 μm filters, followed by 0.22 μm filters, and ultracentrifuge at 100,000× *g* for 70 min at 4 °C, using the SW28 Ti rotor, to concentrate EVs. As these vesicles contained EVs of mouse and human origin, we utilized a commercial human-specific pan-exosome isolation kit (Miltenyi Biotec, San Francisco, CA, USA; Cat 130-110-912), as per manufacturer’s guidelines. The kit was based on the immunomagnetic separation of EVs markers, CD9, CD63, and CD81 expressed on human-specific EVs. EVs obtained from ultracentrifuge step were further separated from mouse liver derived EVs, utilizing Exosome Isolation Kit Pan, human (Miltenyi Biotec, San Francisco, CA, USA; Cat 130-110-912), as per manufacturer’s guidelines. Briefly, EVs resuspended in 1 mL of PBS were incubated with magnetic microbeads that recognizes the tetraspanin proteins CD9, CD63, and CD81of human origin and further passed through a μ column in the magnetic field of a μMACS™ Separator. The magnetically labeled human-EVs are retained within the column, while the unlabeled mouse run through the column. Besides, to confirm human specificity, we used livers from non-humanized mice as negative controls. This strategy is summarized in Supplementary Materials Figure S2a.

### 2.6. Isolation of EVs from Serum

Serum collection and EV isolation from blood were done according to guidelines and methodology published previously [21,23,24]. Briefly, blood was collected in serum tubes (BD Vacutainer, Franklin Lakes, NJ, USA) from treated and untreated humanized mice and non-humanized mice (negative control). Commercial human pooled serum was used as a positive control for human-specific antibodies in the immunomagnetic separation method. Blood was centrifuged at room temperature at 2500× *g* for 15 min, to collect serum and subsequently underwent another centrifugation step at 2500× *g* for 15 min in a clean plastic tube. We used an equal volume (~400 μL) of serum, to isolate EVs by ultracentrifugation. The separation of human-specific EVs from mouse origin EVs was achieved by utilizing a commercial human pan-exosome isolation kit (Miltenyi Biotec Inc, San Francisco, CA, USA), as described in Section 2.5. The strategy is summarized in Supplementary Materials Figure S2b.

### 2.7. Nanoparticle Tracking Analysis (NTA)

EVs isolated from PHH were subjected to NTA, using a NanoSight NS300 (Malvern Instruments Ltd.; Malvern, UK) with a 405 nm and 60 mV laser source as previously described [25]. Typically, 1 mL of a diluted EVs preparation was used for the laser light scattering study. The instrument was set to record at least three recordings per sample at 23 °C for 60 seconds each; NTA software was then used to determine the EVs’ size distribution. For immunomagnetic separation of EVs, EVs were probed with human EVs specific cocktails and passed through the magnetic column, to get EVs conjugated with beads. As the instrument is sensitive to measure any added particles, we separated beads from EVs by disrupting antigen–antibody interaction utilizing low pH (pH 2.8) 0.1 M glycine-HCl and passed them through the new magnetic column. EVs were eluted out with the plunger and the beads retained in the magnetic field of the column. Eluted sEVs were used for counting. The equivalent volume of immunomagnetic beads without EVs incubation served as a positive control for bead retention in the column and negative control for elution of sEVs in eluted buffer. EVs from non-humanized mice and human serum were used as negative and positive controls for showing the specificity of antibodies cocktail to human origin.

### 2.8. ZetaView Tracking Analysis

NTA was performed by using ZetaView Nanoparticle Tracking Analyzer (Particle Metrix, Inning am Ammersee, Germany) and its corresponding software (ZetaView 8.04.02 SP1), to analyze EVs isolated from RLW_XP-GFP_ samples, as published previously [23].

### 2.9. Atomic Force Microscopy (AFM)

AFM imaging of the EVs was performed with the use of instruments at the Nanoimaging core facility at the University of Nebraska Medical Center. Samples were processed to AFM, as described previously with some modifications [23,26]. Briefly, concentrated EVs from RLW_XP-GFP_ cells were diluted in PBS buffer, and 10 μL were deposited onto the piece of freshly cleaved mica. After 2 min incubation, samples were rinsed briefly with several deionized water drops and dried with a gentle flow of argon. Images were collected with the MultiMode Nanoscope IV system (Bruker Instruments, Santa Barbara, CA, USA) in tapping mode at ambient conditions. Silicon probes RTESPA-300 (Bruker Nano Inc., Goleta, CA, USA) with a resonance frequency of ~300 kHz and a spring constant of ~40 N/m were used for imaging at scanning rate for about 2.0 Hz. Images were processed by using the FemtoScan software package (Advanced Technologies Center, Moscow, Russia).

### 2.10. Transmission Electron Microscopy (TEM)

EVs derived from AGS–HIV-treated RLW cells were isolated as described above, and the sample was submitted to the University of Nebraska Medical Center Electron Microscopy Core Facility, to undergo microscopy by a FEI Tecnai G2 Spirit transmission electron microscope.

### 2.11. Western Blot Analysis

EVs derived from each fraction (F1–F12) of cells were lysed in RIPA lysis buffer and processed for immunoblotting as described previously [23]. Total protein was electrophoresed in 4–15% precast protein gels (Bio-Rad, Los Angeles, CA, USA) under reducing conditions followed by transfer to PVDF membranes. Blots were probed with primary antibodies associated with sEVs markers—Alix, TSG10, CD9; non-sEV marker—calnexin; HIV protein—p24 and lysosome marker—LAMP1; and cathepsin D (CTSD) and endogenous control—β-actin. The secondary antibodies were IR dye fluorochrome to anti-mouse or anti-rabbit (LI-COR, Lincoln, NE, USA) or anti-mouse/anti-rabbit HRP. Images were acquired on Odyssey Imaging System (LI-COR, Lincoln, NE, USA) or on ChemiDoc (Bio-Rad, Los Angeles, CA, USA).

### 2.12. Transcriptome Analysis

Total RNA from treated and untreated PHH was isolated as described previously [5]. mRNA sequencing was performed on Illumina next-generation platform. Gene expression was measured in fragments per kilobase of transcript per million mapped reads (FPKM). An FPKM cutoff of >1 and *p* < 0.05 was applied and was used to assess differential gene expression. Cluster Analysis of differential expression genes was used to estimate the expression pattern of differential expression genes under different experimental conditions and clustering of log10(FPKM + 1) values was performed by using Heatmapper (http://www.heatmapper.ca) [27]. Upstream transcriptional regulators were identified in the Ingenuity Pathway Analysis software (IPA, Qiagen, Carol Stream, IL USA), using core analysis of upregulated and downregulated expressed genes. Top 5 upstream transcription regulators (*p* < 0.01) were filtered based on the most significant *p*-value of overlap with the dataset differentially expressed genes. To show an association of upstream regulators with oxidative stress, lysosome and EVs biogenesis, IPA upstream regulator analysis and functional network analysis was used.

### 2.13. Activities of Cathepsins

Cathepsin B and L activities were assayed fluorometrically, as described previously [5,28].

### 2.14. Statistical Analyses

Data from at least three independent experiments were expressed as mean values ± standard error. Comparisons among multiple groups were performed by one-way analysis of variance (ANOVA), using a Tukey post hoc test. For comparisons between two groups, we used Student’s *t*-test. A probability value of 0.05 or less was considered significant.

## 3. Results

### 3.1. Ethanol Stimulates EV Release from HIV-Infected Hepatocytes

**Figure 1:**
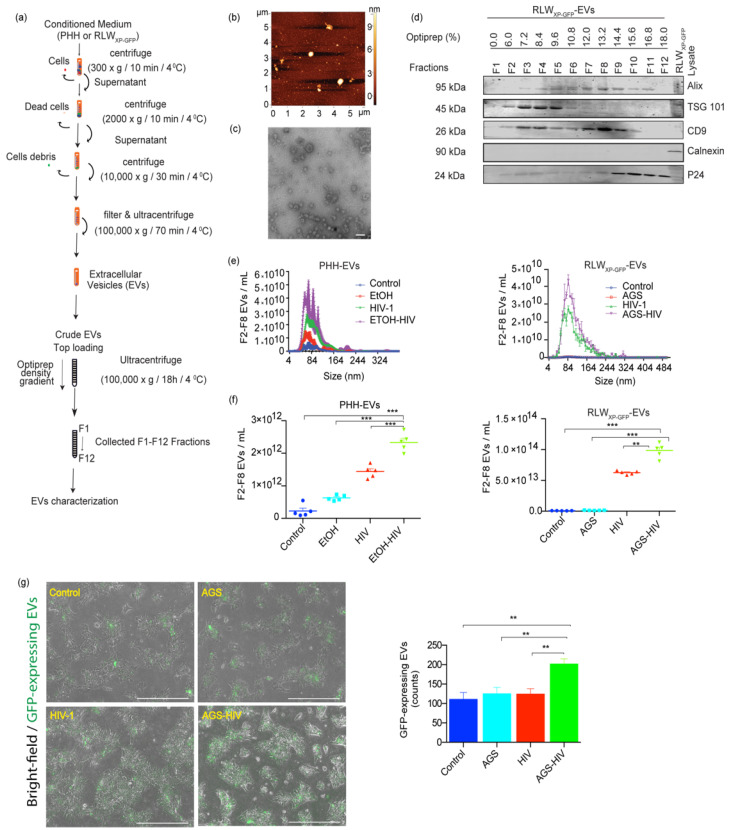
Ethanol (EtOH) stimulates EV release from HIV-infected hepatocytes. (**a**) Schematic demonstration of the process of extracellular vesicles (EVs) separation and OptiPrep density gradient method. (**b**) Topographic profiling of EVs, using atomic force microscopy (AFM) reveals a heterogeneous population of spherical particles of RLW_XP-GFP_ cells. (**c**) Transmission electron microscopy of EVs derived from acetaldehyde-generating system (AGS)–HIV-infected RLW cells reveals a heterogeneous population of small EVs (<200 nm). Magnification 110,000× and scale bar 100 nm. (**d**) Representative immunoblotting images from AGS–HIV treated group show protein expression from F1-F12 fraction of EVs and RLW_XP-GFP_ cells lysate for EV-associated proteins ALIX, TSG101, and CD9, small extracellular vesicle (sEV) negative marker calnexin, and HIV protein p24 enrichment in fractions F9-F12. (**e**) The size distribution of pooled EVs from F2–F8 fractions, released from primary human hepatocytes (PHH) EVs and RLW_XP-GFP_-EV by NanoSight and ZetaView, respectively. (**f**) Dot plots show the concentration of EVs released from EtOH treated HIV-infected PHH and acetaldehyde-generating system (AGS)-treated HIV-infected RLW_XP-GFP_ cells EV by NanoSight and ZetaView, respectively. (**g**) AGS–HIV treatment in RLW_XP-GFP_ cells induces more endogenous EVs, as compared to their respective controls. The bar graph shows quantification of EVs with Fiji software by counting GFP expressing EVs from five to nine randomly captured images with a fluorescence microscope at 10× magnification. Results represent the mean ± SEM. (**e**–**g**) Statistical significance between groups is indicated by asterisk(s) and determined by one-way ANOVA (*** *p* ≤ 0.001 and ** *p* ≤ 0.01).

PLWH are more likely to abuse alcohol [29]. Studies from the past few years have implicated aberrant biogenesis of EVs in the pathogenesis of various alcoholic liver diseases and people with chronic HIV infection [6,30]. Given that hepatocytes represent the majority of cells in the liver and are the primary site of ethanol metabolism, we sought to investigate the effects of alcohol and HIV on EVs release from hepatocytes. PHH and RLW_XP-GFP_ cells were pre-exposed to EtOH and AGS, respectively, followed by HIV infection, as described in Materials and Methods. Alcohol exposure in hepatocytes has been shown to increase HIV expression previously [5]. Conditioned medium was used to separate and concentrate EVs with the OptiPrep method, as shown in the schematic Figure 1a. Isolated EVs demonstrated heterogeneous topography of EVs as assessed by AFM under the tapping mode by immobilizing vesicles on positively charged APS-mica surface (Figure 1b). TEM analysis revealed a heterogeneous population within the expected size range of sEVs (<200 nm) with spherical morphology (Figure 1c). Purification of EVs from HIV is required due to a similar size range and could be obtained by OptiPrep density gradient. Immunoblotting of EV fractions collected from density gradient preparation demonstrated the enrichment for EVs proteins, Alix (F3–F11), TSG 101 (F2–F8), CD9 (F2–F10), and was negative for endoplasmic reticulum protein, calnexin. HIV-specific protein P24 was enriched in fractions F9–F12 in OptiPrep fractions (14.4–18%) and was excluded from EVs analysis (Figure 1d). Size distribution assessed by ZetaView and NanoSight revealed that more than 90% of particles were distributed in the range of typical sEVs (size < 200 nm). Here, EVs showed peak values ranged from mean ± SE: 87.2 ± 30 nm (control), 86.2 ± 35.6 nm (EtOH), 95.3 ± 37.1 nm (HIV) and 102.9 ± 38.5 nm (EtOH–HIV) groups in PHH; and 122.1± 63.8 nm (control), 122.5 ± 70.8 nm (AGS), 109.9 ± 58.1 nm (HIV) and 106.2 ± 46.4 nm (AGS–HIV) groups in RLW_XP-GFP_ cells (Figure 1e). Combined treatment of EtOH and HIV in PHH and AGS and HIV in RLW_XP-GFP_ cells significantly increased the EVs concentration, as compared to their respective controls (Figure 1f). Moreover, endogenous quantitation of GFP-labeled endosomal EVs in the RLW_XP-GFP_ cells revealed an increase in the AGS–HIV group, as compared to the respective controls (Figure 1f). Quantification of GFP-expressing EVs in RLW_XP-GFP_ from five to nine randomly captured images was performed by using Fiji and expressed as average absolute counts.

### 3.2. Hepatocyte-Derived EVs Are Enriched in the Serum of Ethanol-Fed and HIV-Infected Humanized Mice

**Figure 2:**
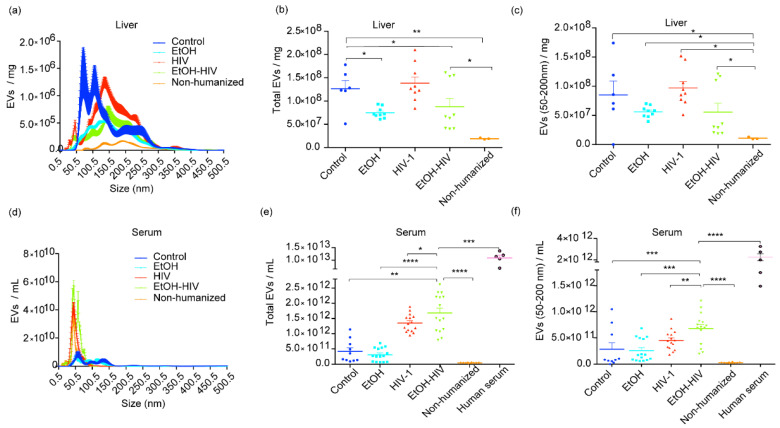
Hepatocyte-derived EVs in the liver and serum from liver-humanized and non-humanized mice. (**a**) The size distribution of hepatocyte-specific EVs for the size range 0.5–500.5 nm in the liver from humanized control, EtOH, HIV-1 and EtOH–HIV, and non-humanized mice. (**b**) Graph showing the concentration of total human hepatocyte specific EVs obtained from the livers. (**c**) Graph showing the concentration of EVs in the size range of sEVs (<200 nm) obtained from the livers. (**d**) The size distribution of human hepatocyte specific EVs in the serum. (**e**) Total human hepatocyte specific EVs in the serum. (**f**) The serum concentration of human hepatocyte EVs in the size range of sEVs. The single dot represents the concentration of EVs in one capture in NanoSight. Equal amounts of livers and serum from untreated and treated mice were used as the starting material to isolate total EVs. Non humanized mice served as a negative, and human serum as positive to the human-specific EVs. All conditions and dilutions were kept identical for all the treatment groups. NanoSight NS300 was used to assess concentration after adjusting for dilutions. Data for the control group were derived from the single control mouse and two pooled control mice. Non-humanized mice data were derived from pooled samples of three mice. Statistical significance between treatment groups is indicated by asterisk(s) and determined by one-way ANOVA (**** *p* ≤ 0.0001, *** *p* ≤ 0.001, ** *p* ≤ 0.01, and * *p* ≤ 0.05).

Based on the premise that EVs biogenesis is altered in alcoholic liver disease and PLWH, we studied whether exposure to EtOH and HIV affects EVs release in humanized FRG-KO mice. FRG-KO mice were either fed EtOH or control diet and infected with HIV-1, as mentioned in Materials and Methods. The specificity of the human pan-exosome isolation kit for EVs of human origin was confirmed by using EVs from non-humanized mice and human serum as negative and positive controls, respectively. Here, human-specific EVs were detected only in humanized mice transplanted with human hepatocytes. Size distribution assessed by NanoSight revealed that more than 75% of EVs were distributed in the range of typical sEVs size of <200 nm (Figure 2a). The results from NanoSight in EtOH–HIV group of mice revealed no statistical difference in the number of total hepatocyte-specific EVs with mean value 8.786e + 007 EVs/mL (range 3.980e + 007–1.610e + 008) vs. EtOH mean value 7.486e + 007 EVs/mL (range 6.090e + 007–9.330e + 007); and HIV mean value 1.384e + 008 EVs/mL (range 8.430e + 007–2.100e + 008) (Figure 2b). EtOH–HIV group-derived EVs in the size range of sEVs (<200 nm) and mean value of 5.569e + 007 EVs/mL (range 1.860e + 007–1.210e + 008) was also comparable to that of EtOH mean value 5.604e + 007 EVs/mL (range 3.970e + 007–7.000e + 007) and HIV mean value 9.702e + 007 EVs/mL (range 5.100e + 007–1.520e + 008) (Figure 2c). Since the EVs derived from humanized mice also include EVs from mouse hepatocytes (~20% of the liver after humanization), our method based on immunomagnetic separation for human-specific EVs was optimal to characterize the treatment-induced changes.

Based on the premise that EVs are increased in the serum of patients with alcoholic liver disease and in PLWH [13,31], we sought to investigate the combined effect of EtOH and HIV on the release of human hepatocyte-specific EVs to the serum of humanized mice. Size distribution assessed by NanoSight revealed that more than 85% of particles distributed in the range of typical sEVs (Figure 2d). The results from NanoSight in EtOH–HIV group of mice demonstrated statistically significant increase in numbers of total hepatocyte-specific EVs (mean value of 1.684e + 012 EVs/mL (range 7.910e + 011–2.620e + 012)) compared with EtOH group (3.091e + 011 EVs/mL (range 6.730e + 010–6.870e + 011)) and HIV group of mice (1.350e + 012 EVs/mL (9.470e + 011–1.890e + 012)) (Figure 2e). Similarly, human hepatocyte-derived EVs of sEVs size range were significantly higher in the serum of EtOH–HIV group (mean value of 6.795e + 011 EVs/mL (range 1.930e + 011–1.210e + 012)) vs. mean value of EtOH (2.568e + 011 EVs/mL (range 6.080e + 010–6.840e + 011)), and HIV group (4.514e + 011 EVs/mL (1.800e + 011–8.670e + 011)) (Figure 2f).

### 3.3. Alcohol–HIV Treatment Regulates Genes Associated with Lysosome and EVs Release in Hepatocytes

**Figure 3:**
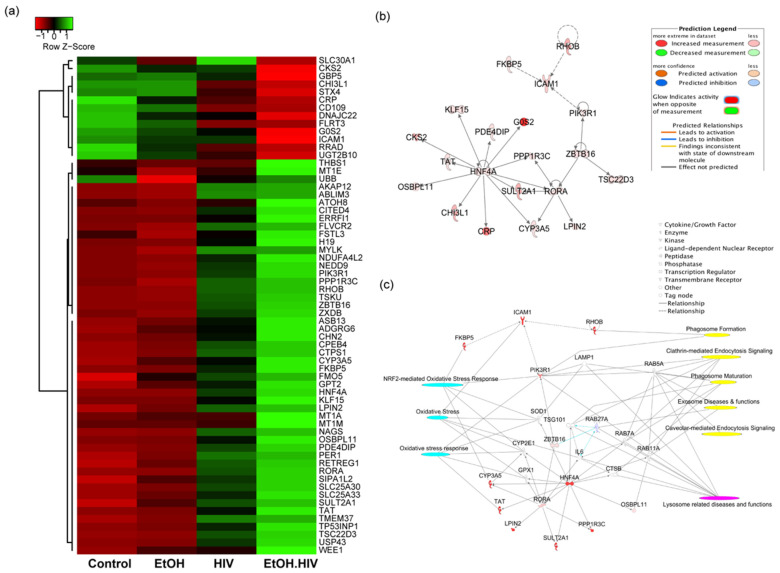
Differentially expressed genes (DEGs) and upstream regulator analysis of primary human hepatocytes and their interaction with target molecules regulating oxidative stress, lysosome related diseases and EVs biogenesis. (**a**) Heat maps and hierarchical clustering-based dendrograms of DEGs from different experimental groups. Expression variability between the groups is indicated by the Z score, where shades of red and green color denote down and upregulation of genes, respectively. (**b**) An integrated network of RORA, ZBTB16, RHOB, HNF4a, and FKBP5 upstream regulators and their target genes. All the targets and upstream regulators were differentially regulated in the dataset based on Ingenuity Pathway Analysis (IPA) upstream regulator analysis. For the color code, refer to the prediction legend. (**c**) Interaction of RORA, ZBTB16, RHOB, HNF4a, and FKBP5 with target genes to regulate oxidative stress (cyan), lysosome-related disease, and functions (magenta) and exosome biogenesis (yellow). Different types of interaction molecules are mentioned in the legend.

We examined the differentially expressed genes in EtOH–HIV treated PHH group, as compared to EtOH and HIV groups and identified upstream regulators that regulate genes associated with EVs biogenesis, oxidative stress, and lysosome-related diseases and functions. Sixty-three (63) differentially expressed genes in the EtOH–HIV group, including upregulated 50 and downregulated 13 genes, were identified by using DEGseq R package, and hierarchical clustering analysis was carried out with the log10 (FPKM + 1) of union differentially expressed genes of all comparison groups (Figure 3a). IPA upstream regulator analysis identified 193 significant upstream regulators (*p*-value < 0.05) in the category of ligand-dependent nuclear receptor (1), transcription regulator (109), kinase (31), and enzyme (52), and out of these categories, five (RORA, ZBTB16, RHOB, HNF4a, and FKBP5) were upregulated in the EtOH–HIV group. Association of these upstream regulators with their targets in the dataset is depicted in Figure 3b. Integration analysis of the upstream regulators with their targets, using toxicity and canonical pathways, revealed dysregulated downstream genes that are involved in the accumulation of oxidative stress (related to genes PIK3R1, FKBP5, CYP3A5, SOD1, CYP2E1, TSG101, GPX1, and TAT1), dysregulation of lysosomal function (CTSB, IL6, Rab27A, TSG101, Rab 7A, Rab11A, PIK3R1, LAMP1, and Rab5A) and EVs biogenesis as evidenced by regulating the pathways associated with phagosome formation (PIK3R1, ICAM1) clathrin-mediated endocytosis signaling (PIK3R1, Rab5A, TSG101, Rab7A, and Rab11A) phagosome maturation (LAMP1, Rab 7A, and Rab11A), and caveolar-mediated endocytosis signaling Rab 5A (Figure 3c).

### 3.4. Alcohol–HIV Treatment in Hepatocytes Causes Oxidative Stress and Downregulates Expression of LAMP1, Which Is Associated with EVs Release

Given the association of EV-associated genes with oxidative stress and lysosomal function in our IPA upstream regulator analysis in the dataset, and as shown previously [13], we sought to check the effects of lysosome impairment on EVs release. Cathepsins B and L constitute the most abundant proteases in lysosomes, and EtOH treatment decreases these enzymes’ activities and is suggestive of lysosomal impairment [28].

**Figure 4:**
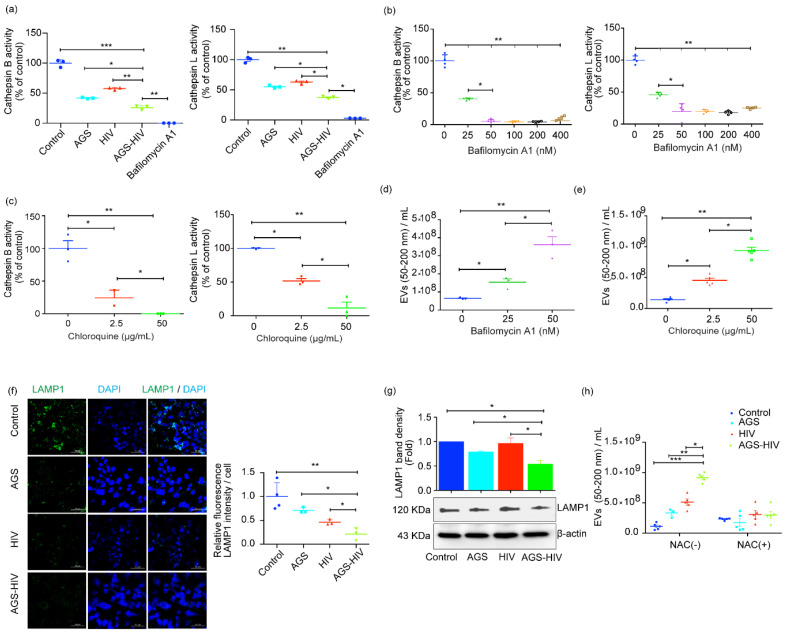
AGS–HIV treatment inhibits lysosomal activities and downregulates LAMP1 in RLW_XP-GFP_ cells. (**a**) Cathepsin B and cathepsin L activities were significantly suppressed by AGS treatment in HIV-infected RLW_XP-GFP_ cells. Bafilomycin A1 was selected to inhibit cathepsin activities and served as a positive control for lysosome inhibition. (**b**) Bafilomycin A1 was used, at different concentrations, to derive gradient in the activities of cathepsins B and L. (**c**) Chloroquine was used at low (2.5 µg/mL) and high concentration (50 µg/mL), to derive gradient in the activities of cathepsin B and cathepsin L. (**d**,**e**) EVs count in the size range of sEVs (50–200 nm) with different concentrations of bafilomycin A1 (**d**) and chloroquine (**e**), as assessed with NTA. (**f**) RLW_XP-GFP_ cells grown on the coverslip and treated with AGS and HIV were probed with LAMP1 antibody, and images were acquired on confocal microscopy. DAPI was used to counterstain the nucleus. For the quantification, Lamp1 intensity was measured from at least three independent images per group and divided by the total number of DAPI positive nuclei. Images were acquired on a 40x objective and scale bar = 50 µm. (**g**) Total protein from whole-cell lysates was extracted from untreated and treated groups and analyzed for lysosome-associated membrane protein (LAMP1) by Western blot. β-actin was used as a loading control. Representative Western blot and densitometric analysis of the LAMP1 level are shown. (**h**) EVs count in the size range of sEVs (50–200 nm) from the cells pretreated with or without the addition of N-Acetyl-L-cysteine (NAC). (**a**–**h**) Data were obtained from at least three independent experiments and presented as Mean ± SEM. Statistical significance between treatment groups is indicated by asterisk(s) and determined by one-way ANOVA (*** *p* ≤ 0.001, ** *p* ≤ 0.01, and * *p* ≤ 0.05).

Thus, we visualized the AGS–HIV-induced lysosomal impairment, and bafilomycin A1 was added as a positive control for lysosome inhibition. AGS–HIV treatment in the RLW_XP-GFP_ cells significantly inhibited cathepsin B and cathepsin L activities, as compared to the AGS and HIV groups (Figure 4a). To further elucidate the crosstalk between impairment of lysosome activity and EVs secretory pathways, we performed a series of cell stimulation experiments. We treated hepatocytes with various concentrations of the drugs bafilomycin A1 and chloroquine, to increase the lysosomal pH, and we established differential levels of cathepsin B and L activities suppression. We observed the concentration-dependent decrease in enzyme activities at 25 and 50 nM for bafilomycin A1 (Figure 4b), and at 2.5 and 50 µg/mL for chloroquine (Figure 4c). The dose-dependent decrease in cathepsin activities was associated with an increase in the sEVs concentration for both drugs, as assessed with the NTA (Figure 4c,d). Given the crucial role of LAMP1 in the interaction of MVBs to the lysosome or phagosome maturation [32], we also checked the expression of LAMP1 protein expression with immunofluorescence and immunoblotting. We observed a significant decrease in the expression of LAMP1 in the AGS–HIV group, as compared to other groups by quantifying images acquired on confocal microscopy (Figure 4e) and in Western blots (Figure 4f). The decrease in cathepsin activities in bafilomycin A1 and chloroquine treated cells showed a significant dose-dependent increase in EVs release, as compared to the untreated control, thereby confirming the lysosome’s role impairment in EVs release. Furthermore, pretreatment with antioxidant NAC in the AGS–HIV group ameliorated the enhanced sEVs release (Figure 4h) and restored lysosomes numbers as assessed with LysoTracker red dye (Supplementary Materials Figure S3), suggesting the role of oxidative stress with the lysosome and in EVs production.

### 3.5. HIV-1 Inhibits Cathepsin B/L Activity, and Lysosomal Dysfunction in the Ethanol-Fed Liver-Humanized Mice

**Figure 5:**
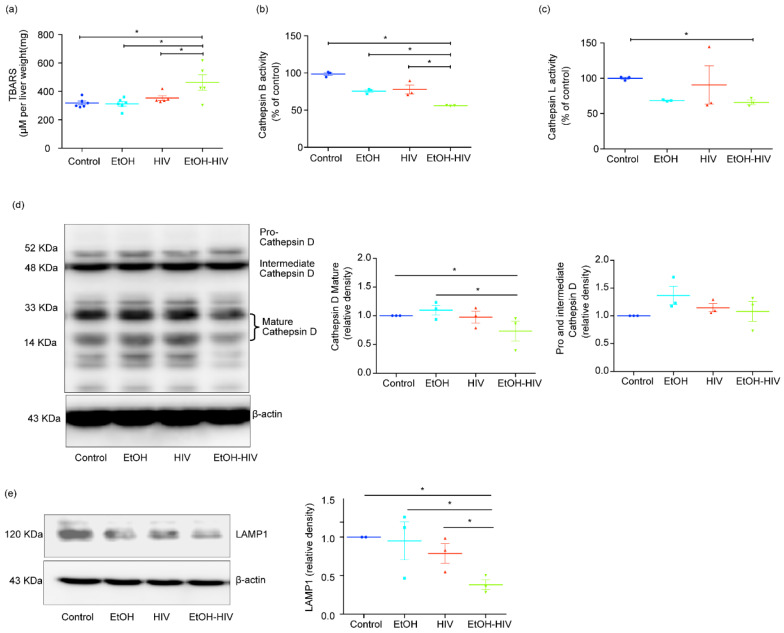
EtOH feeding causes oxidative stress and inhibits the activity of cathepsin B/L and the expression of cathepsin D in the liver of HIV-exposed liver-humanized mice. (**a**) Thiobarbituric acid reactive substances (TBARS) assay. (**b**,**c**) Cathepsin B (**b**) and cathepsin L (**c**) activities significantly suppressed by EtOH feeding in the liver of HIV-infected humanized mice. (**d**) Representative Western blot and densitometric analysis of mature cathepsin D (33 and 14 KDa) and pro–and intermediate cathepsin D (52 and 48 KDa, respectively). (**e**) Representative Western blot and densitometric analysis of LAMP1. (**d**,**e**) β-actin was used as a loading control. Statistical significance between treatment groups is indicated by asterisk and determined by two tailed Student’s *t*-test (* *p* ≤ 0.05).

In hepatocytes, the decline in cathepsin activities by ethanol metabolism has been reported before and was linked to oxidative stress induction [5]. Our lab also demonstrated earlier that EtOH and HIV treatments could induce oxidative stress in the liver in vivo [5]. Similarly, we noticed a significant increase in the activation of oxidative stress in humanized mice’s liver tissues when assessed with the TBARS assay (Figure 5a). In line with our findings in vitro, we also observed significant inhibition in the activity of cathepsin B and cathepsin L in the liver tissue of mice exposed to EtOH and HIV, as compared to HIV and control groups (Figure 5b,c). Cathepsin D maturation is regulated by the activity of cathepsin B and L, so we checked the expression of pro–cathepsin D and mature cathepsin D, produced in the biosynthetic pathway as a precursor pro–cathepsin D (~52 KDa or intermediate cathepsin D ~48 KDa) and sorted to the endolysosomal compartment, where it is processed into a mature form (~34 and 14 KDa) at acidic pH. EtOH-fed and HIV infected mice group showed a significant decrease in mature cathepsin D expression, as compared to control mice (Figure 5d). We observed a significant increase in the EtOH and a trend of an increase in the expression of pro– and intermediate cathepsin D in other treatment groups, which showed no significant difference from mice fed with the control diet (Figure 5d). Besides, LAMP1 expression was significantly downregulated in EtOH-fed HIV-infected mice (Figure 5e).

## 4. Discussion

EVs number and contents are often altered in response to various stresses or pathological stimuli [23,33]. For instance, alcohol has been shown to stimulate EVs release from hepatocytes, microglia, and other cell types [33,34]. HIV induces EVs secretion from various cell types, including (but not limited to) macrophages and neurons [23,35]. Studies from our laboratory also demonstrated that ethanol metabolite, acetaldehyde combined with HIV infection, induced apoptotic bodies from the HIV- and ethanol-exposed hepatocytes, thereby providing detrimental consequences for activation of non-parenchymal cells [5]. However, the release of small EVs from hepatocytes has not been investigated in the settings of HIV and ethanol/ethanol metabolites in the liver cells. Hepatocytes, the primary site of ethanol metabolism, comprise about 80% of liver cells, and cross-talking with non-parenchymal cells can program liver inflammation and fibrosis. In this way, they contribute to the rapid development of end-stage liver disease in alcohol-abusing PLWH. Thus, here, we investigated whether HIV and ethanol/ethanol metabolites modulate EVs release from hepatocytes and, in this regard, which mechanisms are involved in regulating EVs secretion.

In our study, both PHH exposed to ethanol, and RLW_XP-GFP_ cells treated with AGS [36], showed the enhanced EVs release from HIV-infected cells, as compared to cells treated with either alcohol or HIV. Further, no difference in the ratio of EVs to protein concentration demonstrated the purity of EVs (Supplementary Materials Figure S4), suggesting that the increase in the number secreted from the equal number of cells in AGS–HIV treatment group is due to an increase in the EVs/cell, as we consistently observed an increase in EVs’ numbers, as compared to other groups. We have previously shown that hepatocytes could be infected with HIV in vitro and in vivo [5,19]. Most of the EVs (>90%) were sEVs in size range of 50–200 nm. Since the size of sEVs is similar to HIV, it is imperative to separate sEVs from HIV [22,37], which we accomplished by using the OptiPrep fractionation method.

EVs are released to maintain homeostasis, which compensates for the exposure to the stress stimuli that cells can manage without affecting their viability [38]. Hence, when the inclusion of a pan-caspase inhibitor prevented apoptosis in hepatocytes treated with HIV and EtOH and further incubated for an additional three days, we observed the intensive sEVs release. In contrast, hepatocytes exposure to HIV and EtOH in the absence of pan-caspase inhibitor reduced sEVs secretion under the same treatment conditions, suggesting the preferential role of sublethal damage in EVs release (Supplementary Materials Figure S5). Since sEVs number is declined when cells undergo apoptosis and based on our previous results, we observed apoptosis in AGS-pretreated cells in 48–72 h post-HIV exposure [5]. Here, we have chosen a shorter infection duration for EVs studies limited to one day of HIV infection after AGS pretreatment. These conditions were suboptimal for apoptosis but ensured more EVs release.

The EVs release is regulated by lysosome activity in hepatocytes as suppression of enzyme activity with bafilomycin A1 and chloroquine intensifies EVs secretion. Both bafilomycin A1 and chloroquine are shown to impair lysosome activity by lowering the acidic pH (more alkaline) [39,40]. Similarly, AGS–HIV treatment in RLW_XP-GFP_ cells neutralized the lysosomal acidic pH (Supplementary Materials Figure S6) and showed inhibition in lysosomal activities. Thus, the reduction in cathepsin B and L activities is likely associated with decreased lysosome expression (visualized by decreased LAMP1 content) and neutralizing of lysosomal acidic pH. This finding was not surprising since ethanol is known to impair lysosome activity [5], and ethanol-induced EVs secretion from liver cells has already been demonstrated [9,41]. The association of lysosome activity impairment and EVs release was demonstrated in other tissues [41,42]. This study discovered that ethanol-metabolism and HIV-induced suppression of lysosomal activity in hepatocytes increases EVs release from these cells.

In our in vitro model, HIV and ethanol metabolism enhances EVs release under oxidative stress conditions, which are sufficient to induce a level of lysosome damage that does not kill the cells. The NGS study results predict that hepatocytes exposed to HIV and ethanol activated stress-regulated genes and provided lysosome dysfunction in HIV and ethanol-exposed PHH, which potentially increases EVs release from these cells. Our in-silico data revealed that PHH exposed to EtOH–HIV upregulated upstream regulators who regulate genes associated with oxidative stress, lysosomal diseases, and EVs secretion. In our experiments, pretreatment with antioxidant NAC of EtOH–HIV exposed cells restored the lysosomes and suppressed the EVs release, supporting the regulation of EVs release and lysosome by oxidative stress.

To confirm these in vitro findings by in vivo experiments and study in vivo effects of alcohol and HIV on human hepatocyte-derived EVs, we utilized the FRG-KO humanized-liver mouse model [43]. Mice with humanized livers have been used previously for alcohol studies [5,44]. The advantage of this model is that FRG-KO mice livers show robust engraftment of human hepatocytes. Hepatocytes cannot be infected with HIV productively, and HIV infection is abortive [5]. Our previous study also demonstrated that in the absence of immune system, the infection persists for 2 days [19]. Therefore, in the current study, we injected mice with HIV_ADA_ injections every third day. FRG-KO mice in our study showed HIV viral load varying from 394–1486 copies/mL for the HIV group and 350–404 copies/mL in alcohol-fed HIV-infected mice. While EVs in the serum of alcohol-abusing PLWH could come from various organs and subpopulation of cells, and only a part of them is released from hepatocytes, our chimeric liver humanized mice overcome this limitation since the only human cells these mice possess are engrafted human hepatocytes. Thus, the EVs of human origin are hepatocyte-specific. In our study, EVs from serum and livers of humanized mice were separated based on the human-specific pan-exosome markers (a kit from Miltenyi Biotec Inc, San Francisco, CA, USA) as described in Results. Subsequently, we detected the highest level of human EVs in the serum of ethanol-fed and HIV-infected mice, confirming our in vitro findings. Here, we found no difference in HIV–alcohol-induced cell death, which is evident from similar ALT levels in all groups (Supplementary Materials Figure S7). Besides, HIV and alcohol did not cause significant depletion of human hepatocytes as judged by human albumin levels (not shown), but induced oxidative stress in hepatocytes, as evidenced by increased lipid peroxidation (TBARS assay) in the liver tissues.

**Figure 6:**
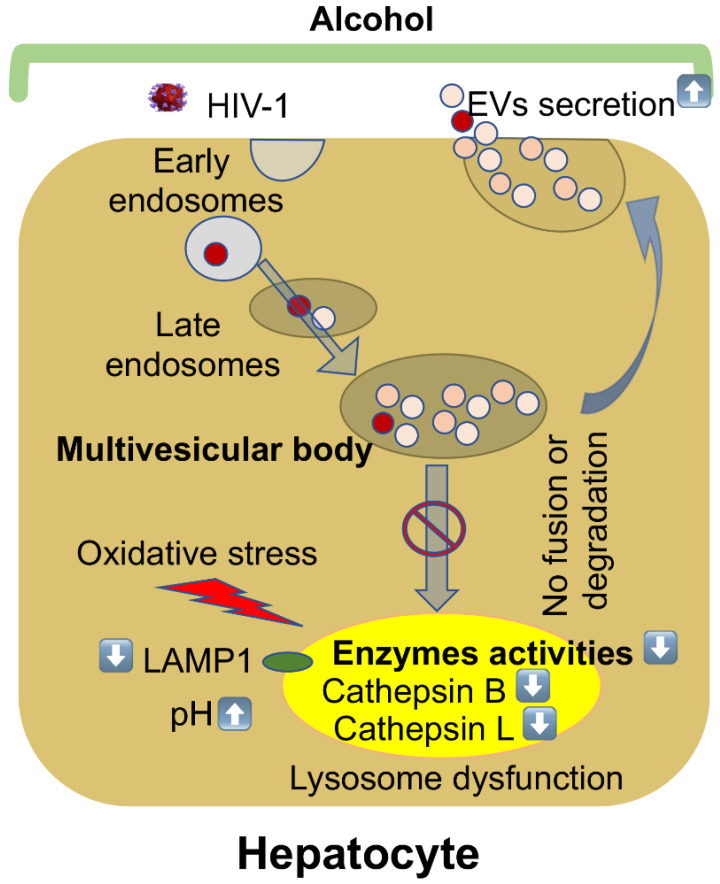
Alcohol stimulates EVs release from HIV-infected hepatocytes by impairing lysosomal function. The maturation of early endosomes results in the formation of late endosomes/multivesicular bodies (MVBs). MVBs that contain many intraluminal vesicles either release EVs by fusion with the plasma membrane, or their contents are degraded if they fuse with lysosomes. Alcohol increases oxidative stress, and lysosomal pH suppresses lysosomal enzyme activities and LAMP1 protein expression in HIV-infected hepatocytes that impair the fusion or degradation of multivesicular bodies to the lysosome, finally leading to an increase in EVs’ release.

We observed a significant increase in the hepatocyte-derived EVs from the serum of mice from the EtOH–HIV group. In accordance to our in vitro findings, in HIV-exposed ethanol-fed mice, there was a reduction in lysosome membrane protein, suppression of cathepsins B and L activities, and inhibition of cathepsin D enzyme maturation in liver tissue, indicating lysosome impairment in mice with the highest levels of hepatocyte-derived human EVs in the serum. The reduction in cathepsin D activity has been shown to cause a significant decline in lysosomal functions [45]. Cathepsin D enzyme is synthesized as an inactive precursor, subsequently processed by cathepsin B and L [46]. Inhibition of cathepsin B and L activities with their specific inhibitors induced accumulation of 48 KDa intermediate cathepsin D by slowing down the cleavage of later into mature two-chain enzyme consisting of a light 14 KDa amino-terminal domain and a heavy 34 KDa carboxyl-terminal domain [46]. Thus, exposure of liver cells to HIV and ethanol metabolites impairs lysosomal function in this organ. Our major findings are summarized in Figure 6.

## 5. Conclusions

In summary, our data demonstrate that ethanol metabolism and mainly acetaldehyde causes oxidative stress in HIV-infected hepatocytes. This results in lysosome impairment associated with decreasing cathepsin activities and downregulation of lysosome protein markers, which, in turn, potentiates EVs release from the hepatocytes. We believe that these results contribute to further understanding underlying mechanisms associated with lysosome impairment and EVs secretion in HIV-induced liver disease progression in alcohol-abusing PLWH.

## Data Availability

Data is contained within the article.

## Supplementary Materials

The following are available online at https://www.mdpi.com/2079-7737/10/1/29/s1. Figure S1: Generation of the stable RLWXP-GFP cell line that produces GFP-labeled EVs, Figure S2: Scheme showing the process of EV isolation, and further purification for human-specific EVs, Figure S3: N-acetyl cysteine (NAC) restores AGS-HIV induced decrease in lysosome. Figure S4: The ratio of EVs and protein concentration isolated from different treatment groups. Figure S5: Apoptosis in RLW_XP-GFP_ cells treated with HIV and EtOH is prevented by pan-caspase inhibitor and restore EVs comparable to control. Figure S6: AGS-HIV treatment neutralizes the lysosomal acidification in RLW_XP-GFP_ cells. Figure S7: Alanine aminotransferase (ALT) levels in the serum of FRG-KO liver-humanized mice were not affected when fed with EtOH in the absence or presence of HIV infection.
