# Supplementary material for: Alcohol-and-HIV-Induced Lysosomal Dysfunction Regulates Extracellular Vesicles Secretion in Vitro and in Liver-Humanized Mice
Source: Biology (Basel). 2021 Jan 5;10(1):29. doi: 10.3390/biology10010029 (PMC7824801; doi:10.3390/biology10010029)
Supplement: Supplementary file 1 [file biology-10-00029-s001.zip › supplementary/biology-1058295-sup.pdf]

Supplementary Materials

Cell viability and cell proliferation assays: Trypan blue dye exclusion assay was performed as described previously [S1]. The effect of lentiviral transduction on cellular proliferation was assessed in RLW and $RLW_{XP\text{-}GFP}$ cells using CyQUANT Direct Cell Proliferation Assay Kit, as per the manufacturer's protocol (Thermofisher; CA, USA) [S2].

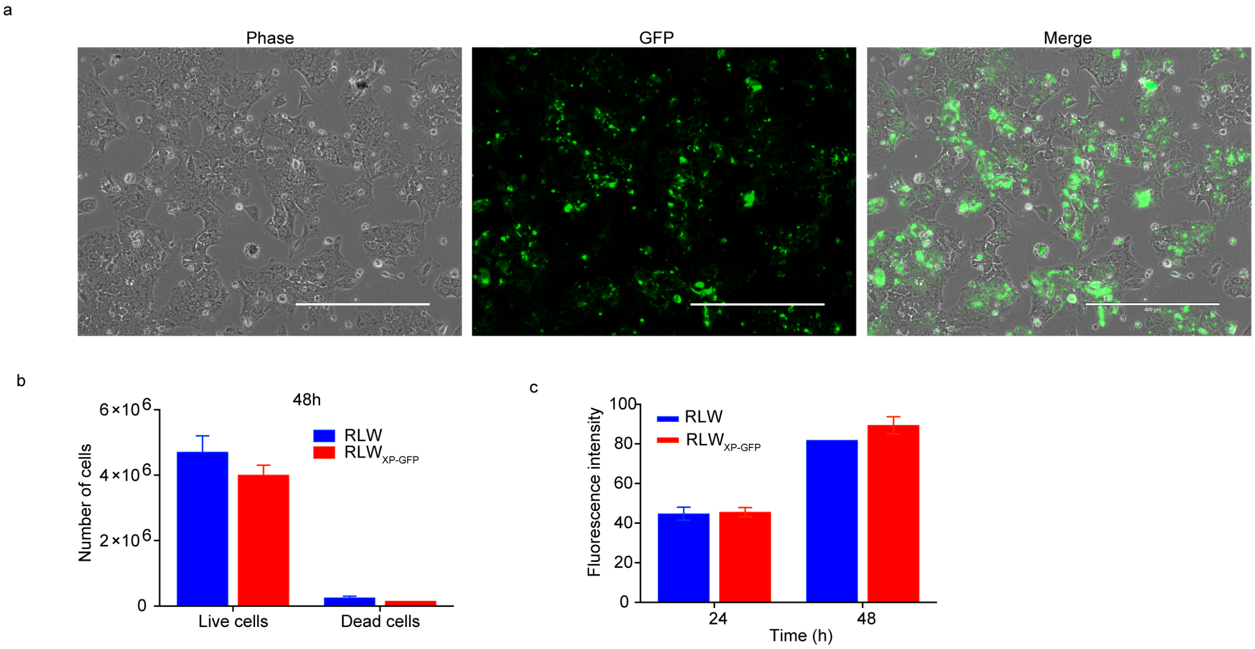

Supplementary Figure S1: Generation of the stable $RLW_{XP\text{-}GFP}$ cell line that produces GFP-labeled EVs. (a) RLW cells transduced with XPack CMV-XP-GFP-EF1α-Puro expression lentivector, following the manufacturer's instruction (XPAK530PA-1, System Biosciences, CA, USA). Following transduction, cells were selected for puromycin- resistance, and then $RLW_{XP\text{-}GFP}$ cells were imaged on fluorescence microscopy for GFP expression. Scale bar = 400µm (b, c) Stable transduction of cells with lentivector did not affect the viability and proliferation kinetics of $RLW_{XP\text{-}GFP}$ from parent RLW cells, as assessed with trypan blue exclusion assay and (c) CyQUANT direct cell proliferation assay, respectively.

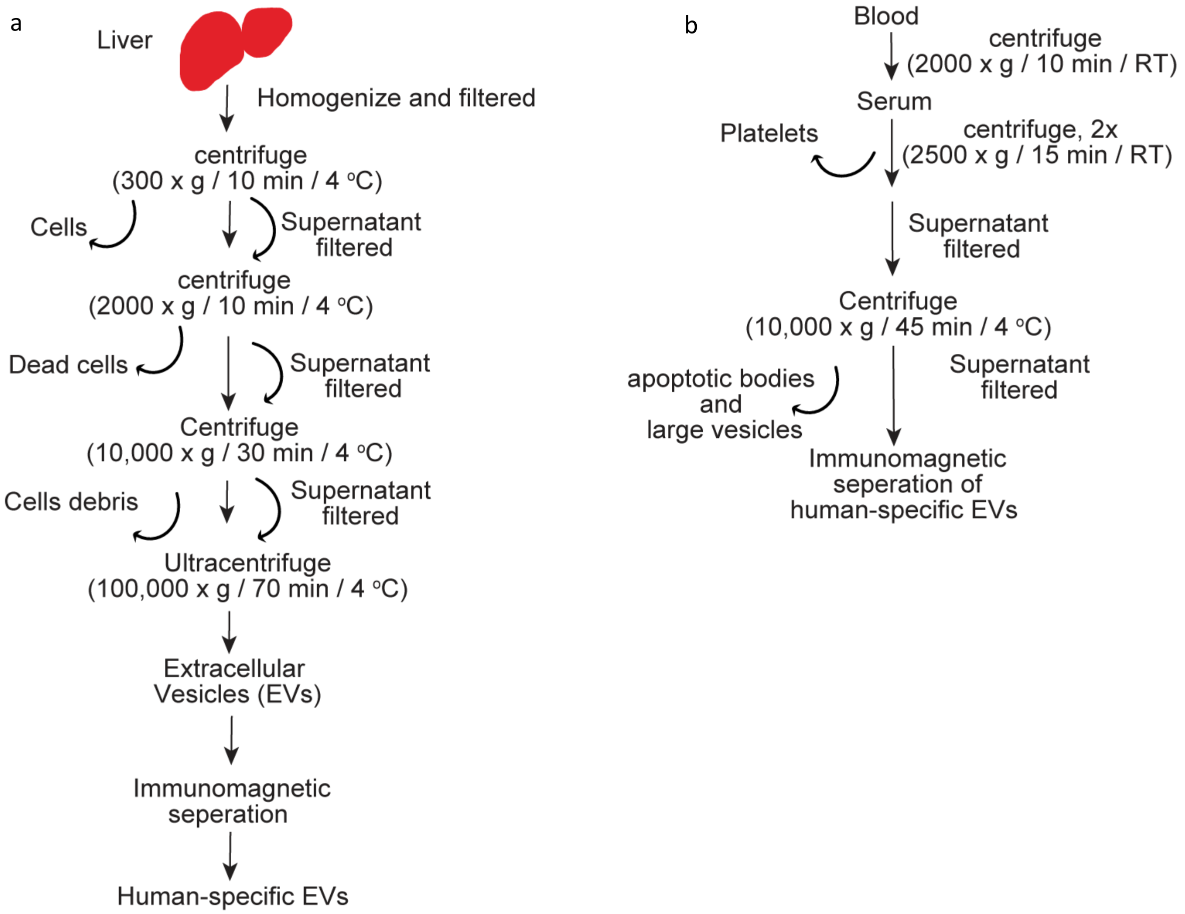

Supplementary Figure S2: Scheme showing the process of EV isolation, and further purification for human-specific EVs from (a) Livers of humanized mice, and (b) serum from humanized mice with immunomagnetic separation using commercial human pan-exosome isolation kit.

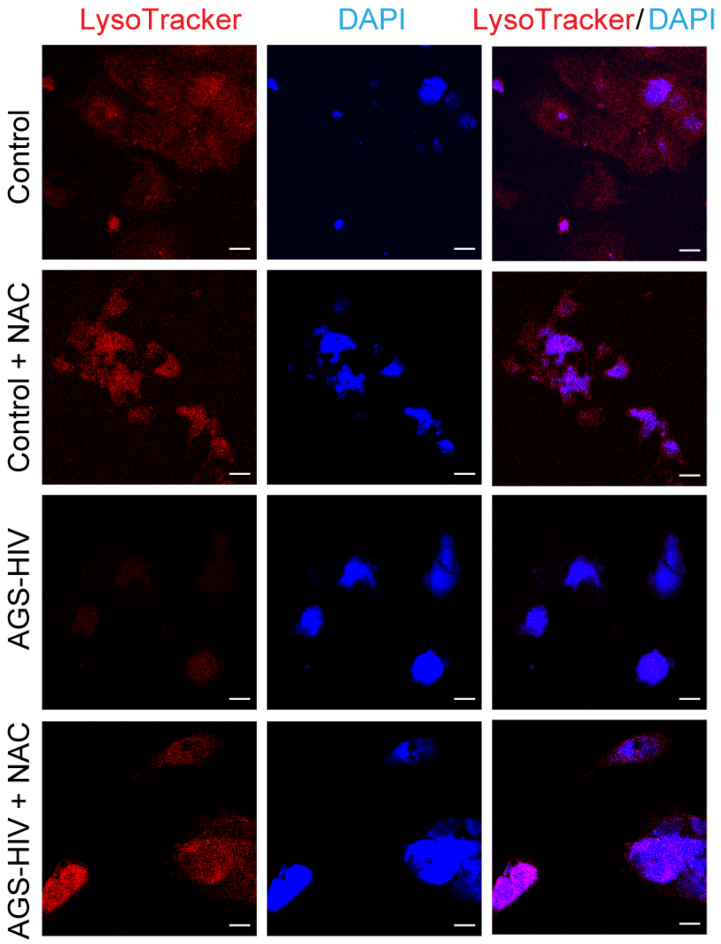

Supplementary Figure S3: N-acetyl cysteine (NAC) restores AGS-HIV induced decrease in lysosome. Pretreatment with NAC in RLW$_{XP\text{-}GFP}$ cells restored AGS-HIV induced oxidative stress mediated lysosome reduction as assessed by confocal microscope LSM710 for LysoTracker positive cells. Cells were counterstained with DAPI for the nucleus. Scale bar = 50 μm.

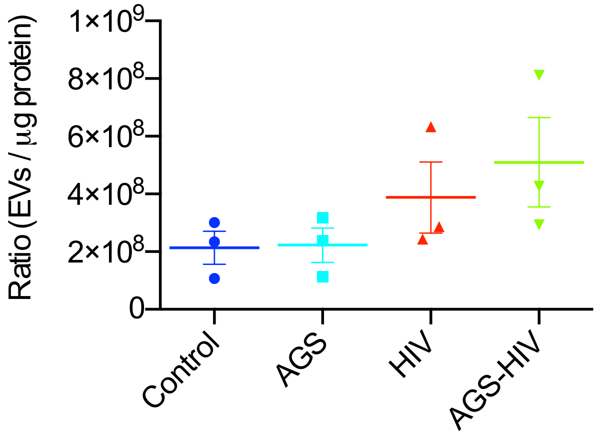

Supplementary Figure S4: The ratio of EVs and protein concentration isolated from different treatment groups. Data is obtained from three replicates and presented as Mean ± SEM. No statistical significance difference was observed within the group using one-way ANOVA.

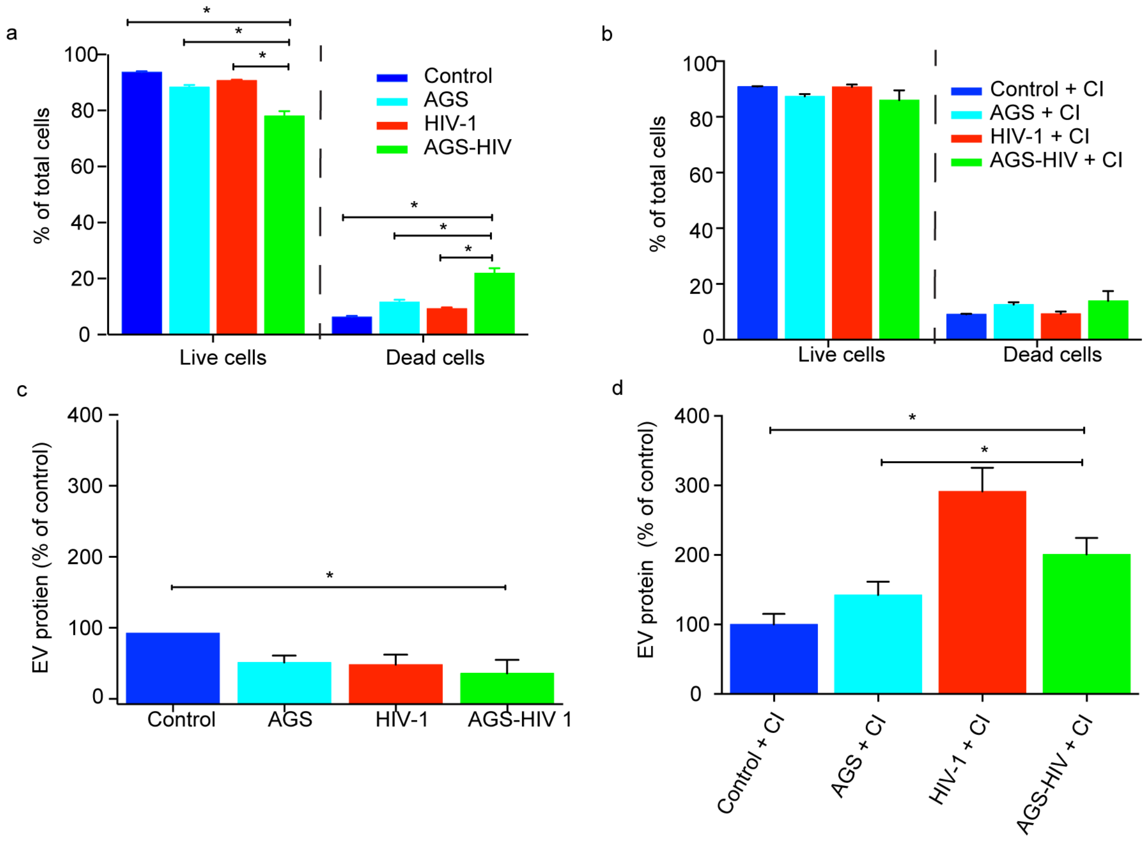

Supplementary Figure S5: Apoptosis in RLWXP-GFP cells treated with HIV and EtOH is prevented by pan-caspase inhibitor and restore EVs comparable to control. The cells pretreated with AGS were infected with HIV for three days. (a-b) Trypan blue exclusion assay for live and dead cells in the (a) absence or (b) presence of pan-caspase inhibitor (CI). Vertical dashed lines separate live cells from dead cells. (c-d) Protein quantification of hepatocyte-derived EVs using bicinchoninic acid assay (BCA) assay in the (c) absence or (d) presence of CI. Results represent the mean± SEM. (a-d) Values are represented as percent of control. Statistical significance for each treatment group vs. AGS-HIV treatment group is indicated by asterisk and determined by two-tailed Student t-test (* $p \leq 0.05$).

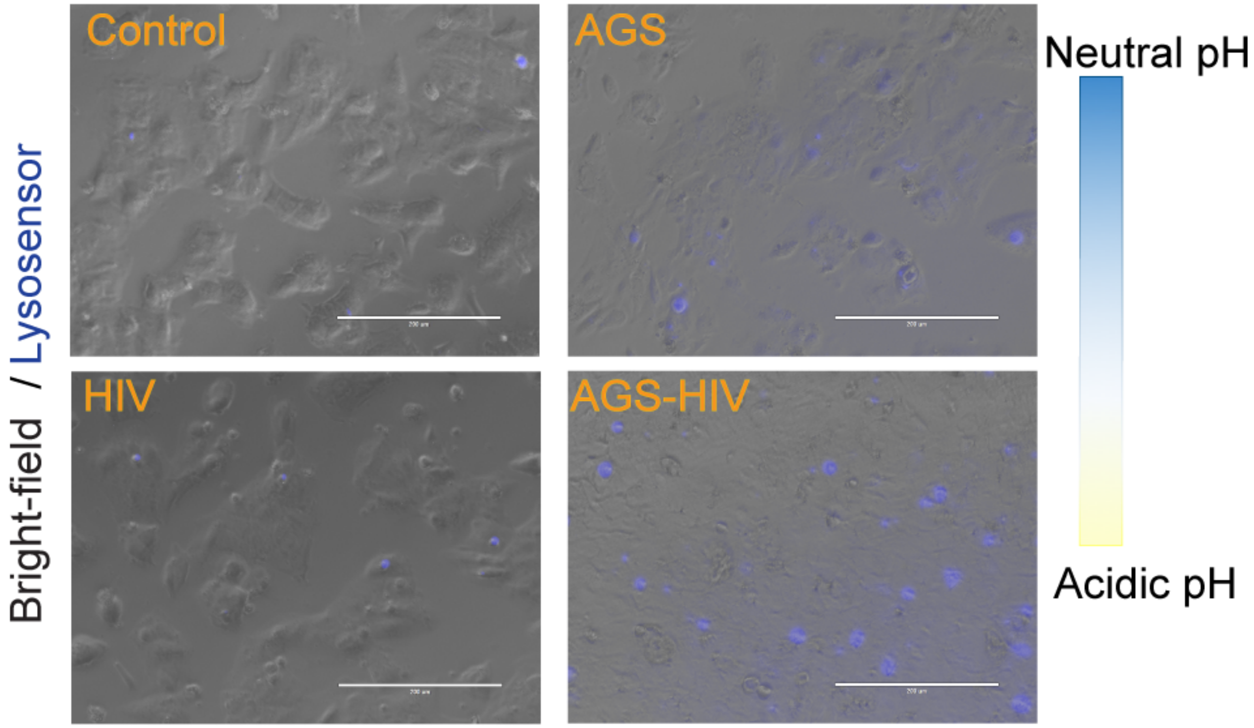

Supplementary Figure S6: AGS-HIV treatment neutralizes the lysosomal acidification in RLW$_{XP-GFP}$ cells. RLW$_{XP-GFP}$ cells infected with HIV in the presence or absence of AGS, stained with 1µM LysoSensor Yellow/Blue DND-160 for 5 minutes at 37°C. After washing with a probe-free medium, the samples were viewed with an EVOS fluorescence microscope (scale bar 200 µm). An increased number of cells exhibiting strong Lysosensor blue signals (indicating neutral pH) were observed in AGS-HIV treated group. An increase in the blue color gradient in the color code bar indicates a more neutral pH.

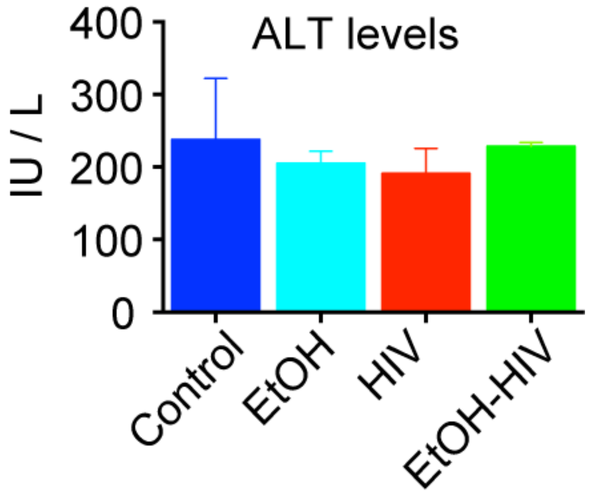

Supplementary Figure S7: Alanine aminotransferase (ALT) levels in the serum of FRG-KO liver-humanized mice were not affected when fed with EtOH in the absence or presence of HIV infection. Results represent the mean± SEM. IU refers to international unit.

Supplementary References

S1 Dagur RS, Hambarde S, Chandna S. Bryostatin-1 causes radiosensitization of BMG-1 malignant glioma cells through differential activation of protein kinase-Cdelta not evident in the non-malignant AA8 fibroblasts. Mol Cell Biochem 2015;401(1-2):49-59.

S2 Tediose T, Kolev M, Sivasankar B et al. Interplay between REST and nucleolin transcription factors: a key mechanism in the overexpression of genes upon increased phosphorylation. Nucleic Acids Res 2010;38(9):2799-2812.
