# Supplementary material for: Alcohol-and-HIV-Induced Lysosomal Dysfunction Regulates Extracellular Vesicles Secretion in Vitro and in Liver-Humanized Mice
Source: Biology (Basel). 2021 Jan 5;10(1):29. doi: 10.3390/biology10010029 (PMC7824801; doi:10.3390/biology10010029)

1.Full western blot images related to figure 1d

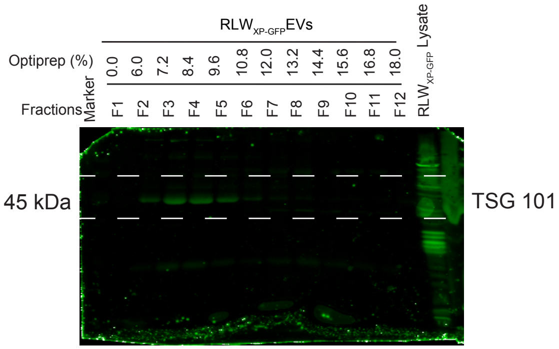

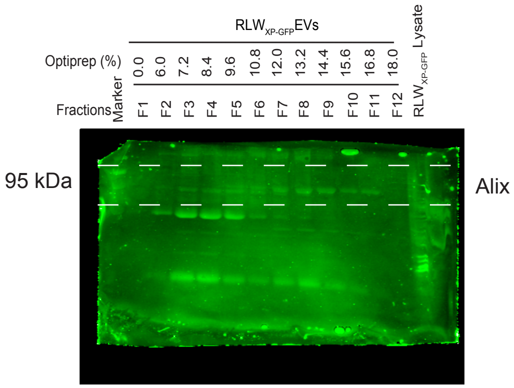

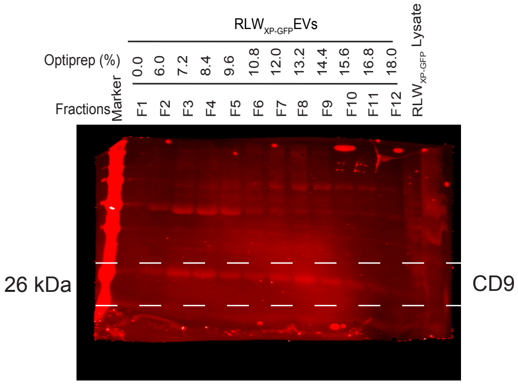

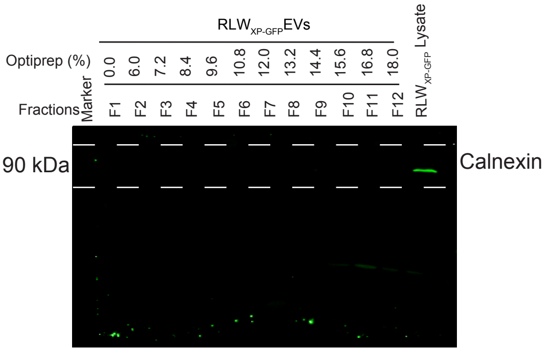

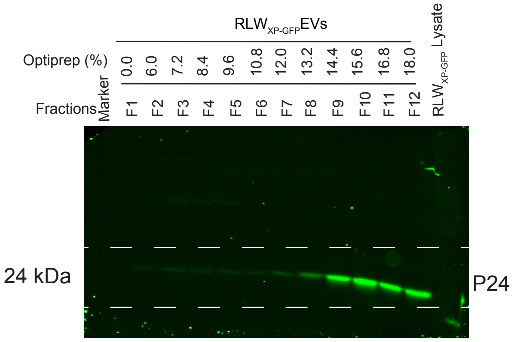

2. Raw western blot images related to figure 4f: Images were captured on Licor imaging system (top) and bio-rad chemidoc system. White dotted box represents the proteins of interest and red dotted box represents the representative blot.

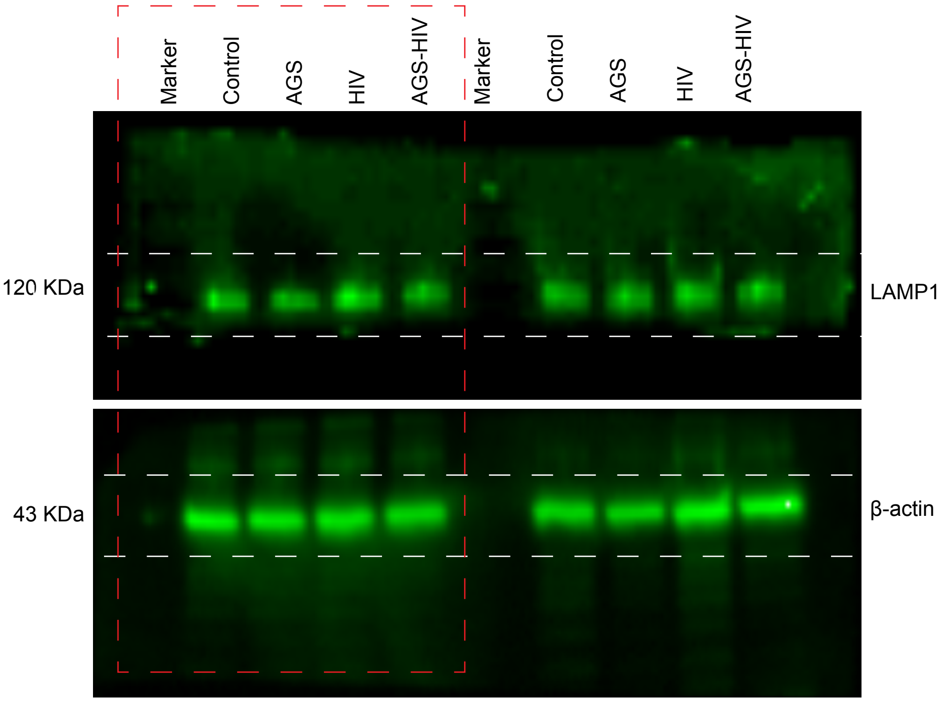

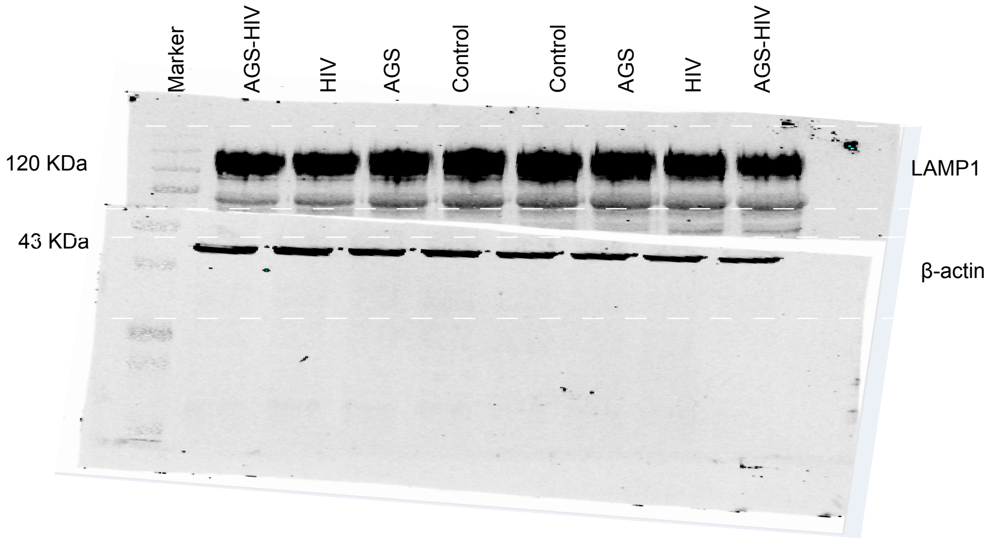

3.**Western blot images related to figure 5d**: Images were captured on Bio-Rad chemidoc system and red dotted box represents the representative blot and black box represent protien of interest

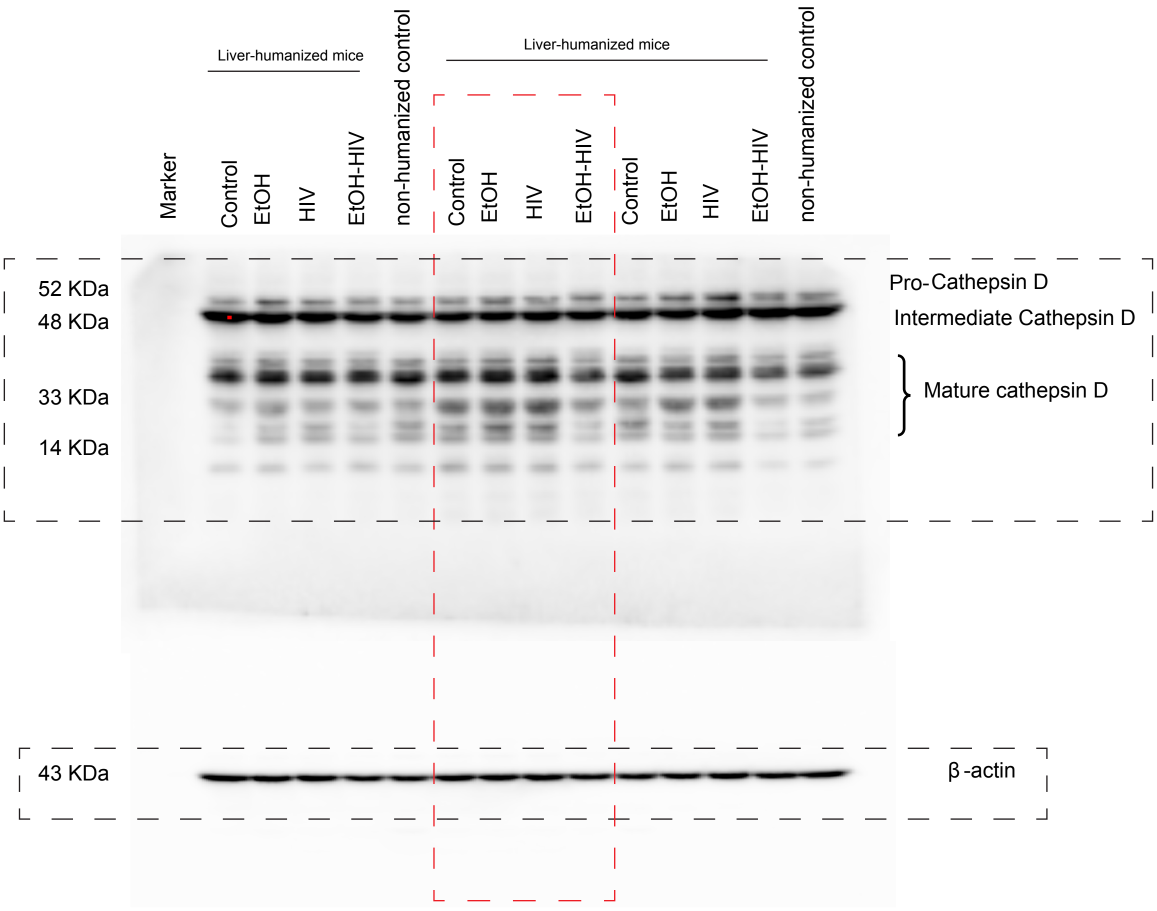

4.**Western blot images related to figure 5e**: Images were captured on Bio-Rad chemidoc system and red dotted box represents the representative blot and black box represent protein of intereest.

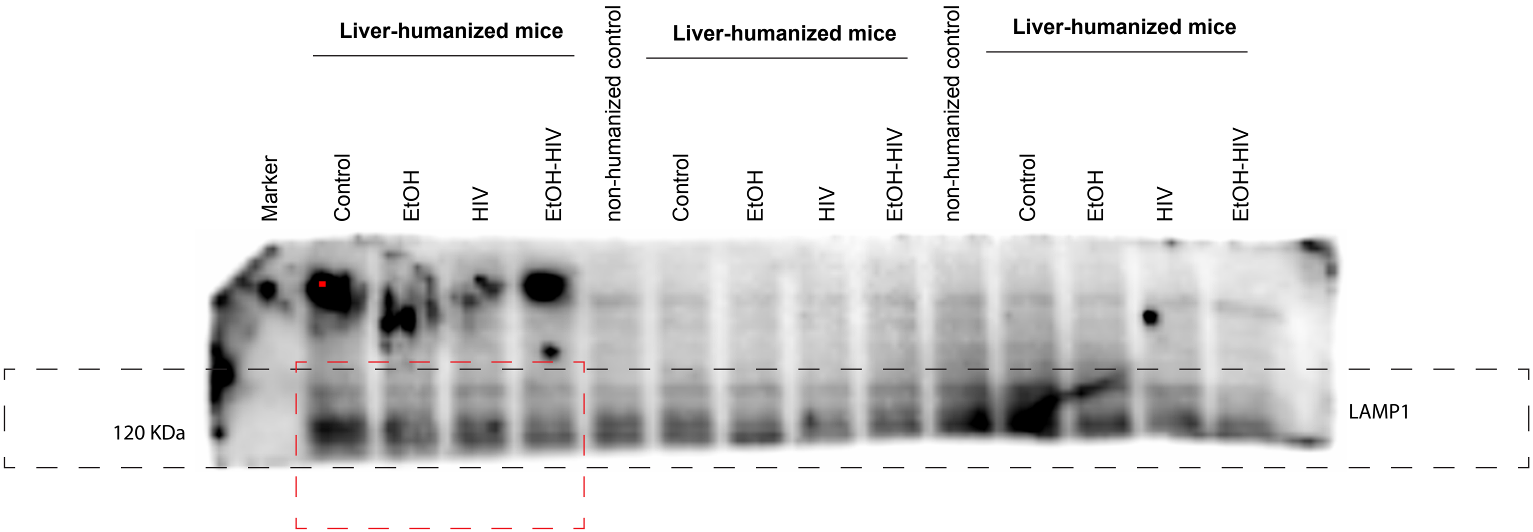

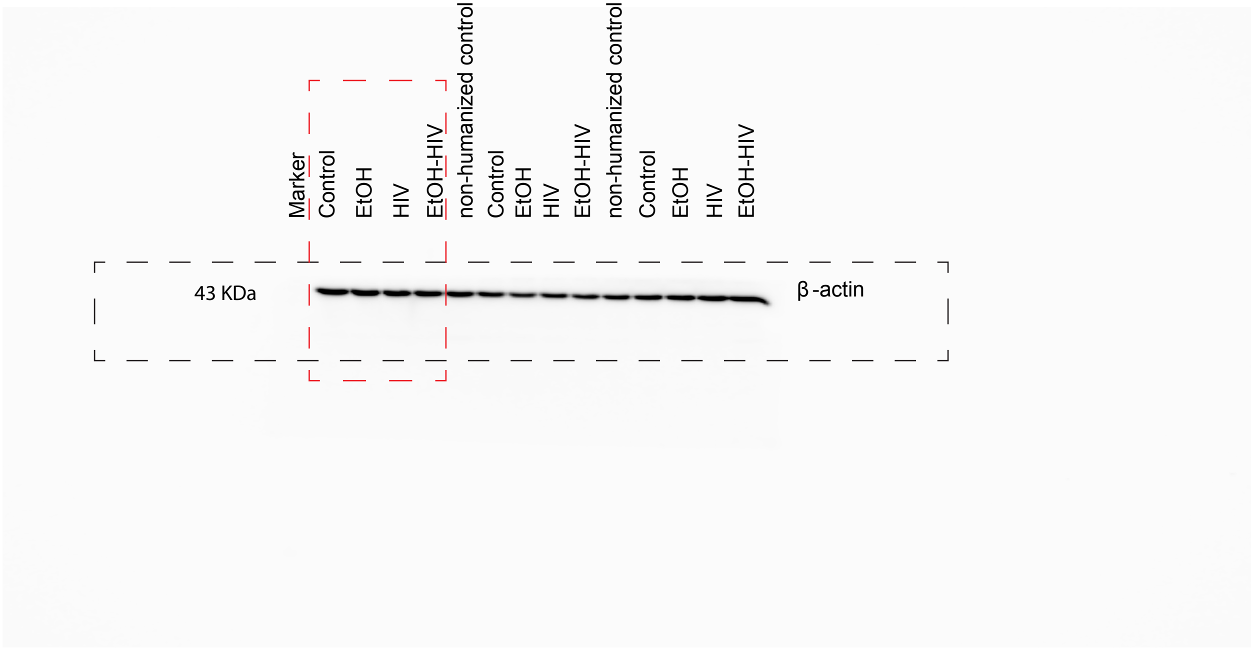

Supplement: Supplementary file 1 [file biology-10-00029-s001.zip › supplementary/biology-1058295-WB.pdf]
